# Primary Cilia as a Possible Link between Left-Right Asymmetry and Neurodevelopmental Diseases

**DOI:** 10.3390/genes8020048

**Published:** 2017-01-25

**Authors:** Andrey Trulioff, Alexander Ermakov, Yegor Malashichev

**Affiliations:** 1 Department of Vertebrate Zoology, Faculty of Biology, Saint Petersburg State University, Universitetskaya nab., 7/9, Saint Petersburg 199034, Russia; trulioff@gmail.com (A.T.); ermakov99@mail.ru (A.E.); 2 Laboratory of Molecular Neurobiology, Department of Ecological Physiology, Institute of Experimental Medicine, ul. Akad. Pavlov, 12, Saint Petersburg 197376, Russia

**Keywords:** schizophrenia, centrosome, left-right asymmetry, Disc1, PCM-1, pericentrin, abelson helper integrator 1, hamartin, DCDC2, Dyx1c1

## Abstract

Cilia have multiple functions in the development of the entire organism, and participate in the development and functioning of the central nervous system. In the last decade, studies have shown that they are implicated in the development of the visceral left-right asymmetry in different vertebrates. At the same time, some neuropsychiatric disorders, such as schizophrenia, autism, bipolar disorder, and dyslexia, are known to be associated with lateralization failure. In this review, we consider possible links in the mechanisms of determination of visceral asymmetry and brain lateralization, through cilia. We review the functions of seven genes associated with both cilia, and with neurodevelopmental diseases, keeping in mind their possible role in the establishment of the left-right brain asymmetry.

## 1. Introduction

Cilia are thin, hair-like structures, projecting from the surface of eukaryotic cells and covered with the cell membrane. Their usual length is 3−15 μm. The cilium consists of the ciliary part (the axoneme protruding from the cell), the basal body, and the transitional zone between the two. The inner part of the basal body serves to maintain its assembly and anchoring.

There are two types of cilia: motile cilia and primary cilia. The functions of motile cilia include cell locomotion and the movement of the fluid surrounding the cell, while primary cilia serve mostly as chemo-, photo-, and mechanosensors [1]. The fundamental difference between these two types of cilia is the absence of the central pair of microtubules in the primary cilia. Although the terms “primary cilia” and “immotile cilia” are often used as synonyms, primary cilia in the central zones of animal embryos can actually move, but unlike motile cilia, they have a rotational pattern of movement. Both motile and primary cilia are necessary for the development and functioning of the nervous system. Motile cilia are only present in a subpopulation of the cells of choroid plexus and in the ependymal cells of brain ventricles. They are essential for cerebrospinal fluid movement [2,3]; the lack of cilia motility can lead to hydrocephalus [4] and prevent migration of some neural progenitors with the cerebrospinal fluid flow [3]. Primary cilia are found in most types of brain cells: neurons, glial cells, neural stem cells, and some cells of choroid plexus. Specialized primary cilia are found in visual, acoustic-vestibular, and olfactory receptors. Several neuromediator receptors (dopamine receptors 1, 2, and 5, somatostatin receptor 3, and serotonin receptor 6), are expressed in neuronal primary cilia [5]. Moreover, primary cilia act as receptors in morphogen-mediated Shh, and Wnt and Fgf signaling, which are essential for embryonic and adult development of key brain regions, e.g., the hippocampus and cerebellum [6].

More than 1000 ciliary proteins are involved in cilium assembly and functioning [7]. Mutations in their genes lead to defects in cilia, which in turn cause a range of human disorders, referred to as ciliopathies. The first described ciliopathy was the Kartagener syndrome [8,9]. Since then, more than a hundred human disorders have been linked to defects in cilia [7].

Ciliopathies are usually associated with congenital kidney dysfunctions, mental retardation, obesity, hepatic disease, craniofacial defects, retinopathy, polydactyly, and other symptoms. In some cases, ciliopathies are accompanied by situs inversus viscerium. This is due to the fact that cilia are involved in the left-right axis determination [7]. Ciliary beating at the node during the neurula stage leads to the generation of leftward fluid flow, which in turn causes an expression of the genes of the Nodal cascade in the left side of the embryo. Remarkably, the structure of beating cilia in the node is that of primary (9 + 0), not motile (9 + 2), cilia. Since they lack the central pair of microtubules, they have a rotational type of movement, and this rotation produces a left-sided nodal flow.

There are two hypotheses explaining how the fluid flow affects left-sided nodal expression. According to the first theory, the concentration of specific signaling molecules increases in the left side of the embryo due to the fluid flow, and this is sufficient enough to activate the expression of the Nodal signaling cascade [10,11]. The other hypothesis assumes that there are two cilia types in the node: the movable primary cilia, which rotate and generate the fluid flow, and the immotile primary cilia, which serve as mechanoreceptors of the flow; the reception results in a rise of intercellular [Ca^2+^] [12], which leads to activation of the expression of specific “left-sidedness” genes [13]. The latter hypothesis offers a more convincing explanation of how primary cilia breakdowns cause situs inversus. However, evidence from medaka fish raises a possibility for a mixed mechanism, in which the same motile cilia may serve as both a flow motor, and a chemical sensor of a Nodal cascade triggering molecule [14]. Initially, the involvement of cilia in the left-right patterning was shown for mammalian development [10,11], but the same mechanism has since been hypothesized for other vertebrates [15], and for zebrafish [16] and *Xenopus* [17], though not for the chick [14,18]. More recent evidence has suggested that a similar mechanism may be involved in ascidians [19].

Asymmetrical Nodal-cascade gene expression is involved, not only in the formation of the normal visceral situs, but also in the establishment of asymmetry in the zebrafish brain, e.g., habenular and parapineal nuclei [20,21]. Moreover, zebrafish of the mutant line fsi (fsi stands for “frequent-situs-inversus”), often demonstrate a concordance between visceral and brain asymmetries, including the partial alteration of the behavioral lateralization [22]. In addition, *nodal*, *lefty*, and *pitx2* are asymmetrically expressed in the dorsal part of a shark′s brain, but there is no evidence of Nodal cascade participation in the formation of the interhemispheric asymmetry of the telencephalon in mammals [23]. For instance, Broca′s and Wernicke′s areas, responsible for speech in the human and homologous in the ape brain, are located in the left hemisphere, but there is no evidence of a developmental mechanism for this asymmetry. Among people with situs inversus totalis, translocation of speech function from the left hemisphere to the right one is not observed [24]: left-handers are encountered among people with situs inversus as often as among people with situs solitus [25], while the standard dichotic listening test reveals the same results among the people of these two groups [26]. Moreover, language dominance, as revealed by magnetoencephalography and the anatomy of petalia, though not planum temporale, as revealed by magnetic resonance imaging, correlate to situs inversus, suggesting that brain asymmetries might develop via multiple mechanisms [27]. A certain discrepancy between the fish and the human data has not been satisfactorily explained thus far, but since no single gene has been found for the fsi zebrafish line, which would account for the resulting morphological and behavioral phenotypes, it is probable that both genetic and environmental mechanisms are involved. On the other hand, different developmental mechanisms may result in situs inversus of the inner organs with the same morphology, but not necessarily in the same functional asymmetry of the brain [27,28,29,30].

Besides the alterations in visceral left-right asymmetry, defective cilia may also result in the absence of the corpus callosum [6], which plays an important role in the maintenance of interhemispheric crosstalk and functional brain asymmetry, including handedness, bilateral representation of language, functional interhemispheric inhibition, and differences in arousal [31,32]. It is worth noting that an association between relative hand skill and single nucleotide polymorphism (SNP) in a PCSK6 gene, whose product is involved in a Nodal regulation during embryogenesis, was observed in dyslexic patients [33]. However, in the control group, there were no associations that could be explained by epistasis between genes responsible for dyslexia or handedness [33]. Thus, cilia may be involved in different developmental mechanisms, which directly or indirectly influence the asymmetric functions of the human brain, as well as in neurodevelopmental disease. It has been suggested that the lateralization of brain functions confer an evolutionary advantage, by improving the capacity to perform parallel tasks in contralateral brain hemispheres [34]. The left hemisphere is associated with distinguishing various stimuli and focusing attention, whereas the right hemisphere is used to react to danger and express emotions [35]. Therefore, alterations in brain asymmetry can cause impairment in the efficiency of input information processing. A disturbance of the brain asymmetry may correlate with mental disorders: autism [36], dyslexia [37], depression [38], bipolar disorder, and schizophrenia [39,40].

So, on the one hand, cilia defects lead to visceral asymmetry abnormalities, and on the other hand, brain lateralization defects correlate with mental disorders. It is tempting to assume an important role of cilia in the establishment of brain asymmetry, even though there are no direct links between visceral and functional brain asymmetry. There is, however, evidence that visceral asymmetry aberrations, at least in some cases, are comorbid with the disorders listed above: schizophrenia [41,42,43,44], depression (and psychotic disorders) [45], and autism [46]. Since we believe that disturbances in brain lateralization can lead to neurodevelopmental diseases, a method which could be used to search for responsible genes would be to check whether neural lateralization and visceral left-right asymmetry have common underlying genetic mechanisms. The genes involved in vertebrate left-right asymmetry establishment may also participate in the lateralization of certain brain regions in *Danio rerio* [21] and dogfish [23], and eye migration to the side in flatfishes *Paralichthys olivaceus* and *Verasper variegatus* [47]. Asymmetric expression of Nodal-cascade proteins in the brain of developing flatfishes has been correlated with the neuronal architecture associated with the alteration of the position of the eyes and orbits [48]. Since cilia are involved in visceral left-right asymmetry in mammals and some other vertebrates, and disturbances in expression of the cilia genes can lead to abnormal left-right asymmetry (e.g., in primary ciliary dyskinesia), we aimed to test the idea that the functions of cilia do affect brain lateralization, and that disturbances of the ciliary structure and function would cause neurodevelopmental diseases. In this review, we explore possible connections between cilia genes and mental disorders, linked with brain laterality defects.

**Figure 1:**
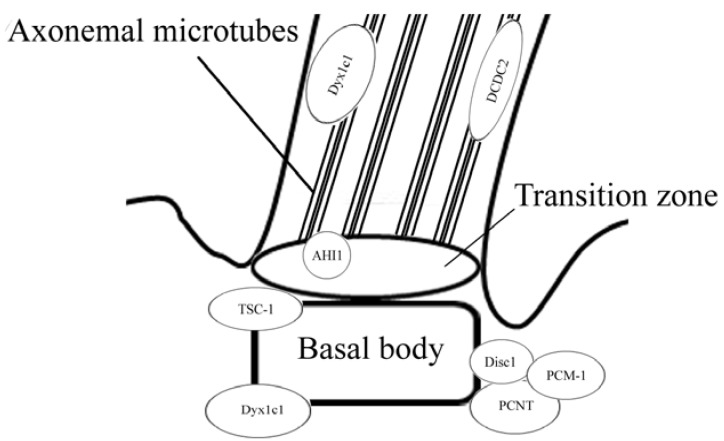
Ciliary proteins localization in a cilium. Most of the proteins are localized in the basal body, AHI1 is localized in the transition zone, and two of the proteins are localized in the axoneme.

We show that proteins associated with the primary cilia may be involved in neurodevelopmental pathogenesis, and in many cases, influence visceral asymmetry (Table 1). Disrupted in schizophrenia 1 (Disc1), pericentriolar material 1 (PCM-1), and human jouberin (Abelson helper integration site 1 (AHI1)) are linked with schizophrenia, hamartin (Tuberous sclerosis 1 (TSC1)) is linked with autism, and pericentrin (PCNT), DCDC2, and Dyx1c1 are linked with dyslexia. It has been shown that genes, which encode these proteins, are expressed in the central nervous system. Most of these proteins (Disc1, PCM-1, jouberin, and hamartin) are localized in the basal body of the cilia (or in the ciliary transitional zone as jouberin), whilst DCDC2 is an axonemal protein, and Dyx1c1 is localized in both the centrosome and the axonemal part of the primary cilia in some cells (Figure 1).

## 2. Disrupted in Schizophrenia 1 (Disc1)

In 1990, a balanced translocation t (1:11) (q43, q21) was described in one Scottish pedigree. Within this family, one third of the family members suffered from mental and/or behavioral disorders [77], including schizophrenia, schizoaffective disorder, and bipolar affective disorder. It was later identified that this translocation resulted in the breakdown of a gene, which was named *disrupted in schizophrenia 1* [78]. More than 10 years of studies focusing on *disc1* in various populations, have established its involvement in a number of psychiatric diseases: autism [79], depression [80,81], bipolar disorder [82,83,84], and schizophrenia [78]. Disc1 has many binding partners and is thus involved in many physiological processes. Its mutations, or a disturbance of its expression, lead to their breakdown.

Within the cell, Disc1 is located in several cell compartments, including the nucleus, centrosome, microtubules, and mitochondria. Disc1 is localized near the base of immotile cilia in the cultured NIH3T3 cells and rat striatal neurons. It is essential for primary cilia formation; knockdown of this gene results in the loss of immotile cilia, and in a decrease of dopamine receptors on the cell surface [49]. A defect in ciliogenesis, caused by mouse *disc1* suppression, could be rescued by human Disc1 in NIH3T3 [49]. Moreover, Disc1 is probably involved in intraflagellar transport regulation, because it interacts with MIPT3 (microtubule-interacting protein associated with TNF receptor associated factor-3) [50], which is crucial in forming intraflagellar transport particle complexes [85].

In addition to its role in the formation of primary cilia, Disc1 is involved in plus-end and minus-end microtubular transport: Disc1 interacts with microtubule motor proteins, dynein intermediate chain (DynIC) and Kinesin-1. Taking into consideration the fact that Disc1 can interact with a wide range of cellular proteins, it has been suggested that Disc1 is required for the transport of various cargoes as an adaptor, and helps to attach them to microtubule motor proteins [86].

During neurogenesis, an accurate position of the centrosome is important for neuronal cell migration and for determining the fate of the daughter cell (i.e., a cell’s decision to be differentiated into a neuron or to remain as a progenitor cell) [87]. In cortical neurons, Disc1 is co-localized with γ-tubulin and is required for the assembly of centriole [88]. Disc1 can interact with multiple proteins of the centrosome, anchoring the dynein motor complex to the centrosome. Based on this fact, it is suggested that *disc1* expression in neuronal cells is crucial for neuronal development and migration. *disc1* knockdown disconnects the nucleus and centrosome during cell migration, which results in an abnormal development of the cerebral cortex [88].

The ability of Disc1 to interact with the intracellular transport proteins makes it a significant factor in the functioning of the nervous system. Disc1 is involved in neurite outgrowth [89], and regulates the structure and functioning of the synapses [90]. Since Disc1 knockdown inhibits microtubule-associated cellular transport of various cargoes, it probably participates in both anterograde and retrograde transport, through the axon [5]. Moreover, Disc1 is a key factor in the development of the central nervous system. Besides regulating neuronal cell migration, it also regulates neural cell proliferation, e.g., cortical progenitors in utero and in adults [91]. In both humans and rodents, *disc1* expression is the highest during the developmental stages of the central nervous system, after which it gradually decreases [92].

Disc1 interacts with girdin [93] and β-catenin [91], and participates in Akt/mTOR, GSK-3/β-catenin, and Wnt signaling, which are all involved in neurogenesis and adult neurodevelopment. Moreover, Disc1 is connected to several pathways of neuronal signal transduction, including PDE4/cAMP [94], GABA [95], and dopamine [49] mediated pathways. Mutations in *disc1* alter the intracellular GABA transport [51,96]. Some of these pathways, e.g., GSK-3/β-catenin, are related to neurodevelopmental disorders, such as schizophrenia [97,98,99].

In sum, Disc1 is involved in the development and functioning of the nervous system and, as an interactor with a set of proteins, is responsible for various neural cell functions. Mutations in *disc1* result in mental disorders, such as autism, depression, bipolar disorder, and schizophrenia. However, these mental dysfunctions are caused by Disc1 deficit, and are not associated with cilia dysfunctions. For example, Disc1 is also located in mitochondria, which are involved in some human diseases of the central nervous system, including schizophrenia and bipolar disorder [52].

## 3. Pericentriolar Material (PCM-1)

Pericentriolar material 1 is a protein, which was initially described in HeLa cells as being associated with the centrosomes in the interphase, and dispersed throughout the cell during the rest of the cell cycle [100]. The gene *pcm-1* is located on chromosome 8. Later, it was shown that PCM-1 is a component of centriolar satellites [101], small non-membranous 70–100 nm particles, which surround the centrosome; similar structures also exist around the basal bodies in ciliated cells. During induced ciliogenesis in murine nasal respiratory epithelial cells, the content of PCM-1 in the apical cytoplasm increased [101]. PCM-1 has coiled-coil domains underlying its ability to undergo oligomerization [102] and interaction with other proteins.

PCM-1 is reported to contribute to the dynein-dependent, microtubule-based trafficking of proteins to the centrosome [55]. It makes complexes with itself, Disc1, and BBS4 (Bardet-Biedl syndrome 4) [56], which can bind cargo proteins to dynein. PCM-1 is crucial for the assembly of the centrosomal proteins centrin, pericentrin, and ninein at the centrosome; the organization of a radial microtubule network depends on PCM-1, and depletion of PCM-1 inhibits anchorage of microtubules to the centrosome [55]. It is worth noting that depletion of the centriolar satellite protein PCM-1 has no effect on centriole assembly, but reduces the amount of centrosomal proteins at basal bodies [103].

PCM-1 is also thought to be critical for flagella assembly. In *pcm-1* zebrafish morphants, the cilia in pronephros were reduced in length, which was correlated with the dose of morpholino used in the experiment. In morphant embryos, cilia within the Kupffer′s vesicle were less than half as long as in controls, which led to inverted heart looping, consistent with randomization of left–right asymmetry [104].

PCM-1 is probably involved in cilia assembly by interaction with proteins such as BBS4, CEP290 [105,106], and OFD1 [107]. Complex PCM-1-CEP290 is pivotal to targeting Rab8 to promote ciliogenesis. In this way, PCM-1 function is required for the formation of the non-motile primary cilium [105]. Also, PCM-1 regulates ciliogenesis through interacting with Htt, whose depletion leads to dispersion of PCM-1 satellites and impairs primary ciliary formation [106].

PCM-1 takes part in cilia disassembly before mitosis. Polo-like kinase 1 (Plk1) promotes primary cilia resorption by activating histone deacetylase 6 (HDAC6), a tubulin deacetylase which is responsible for modulating cell spreading and motility, as well as primary cilia resorption. Along with the latter process, during mitotic G2 phase, Plk1 is accumulated around the pericentriolar matrix, where its recruitment is carried out by PCM-1, phosphorylated by CDK1 [57].

Interestingly, PCM-1 interacts with Disc1, and localization of PCM-1 in the centrosome is regulated by this interaction. An allelic variant of Disc1, which is associated with schizophrenia-related phenotypes, Leu607Phe, and an allelic variant Ser704Cys, affects the PCM-1 distribution in the centrosome [108,109].

In the developing cerebral cortex, suppression of *PCM-1* leads to neuronal migration defects [56]. Population studies in the United Kingdom have demonstrated that *pcm-1* is implicated in susceptibility to schizophrenia [110]. SNP rs370429 in *pcm-1*, which causes the isoleucine to change to threonine, has also been shown to be associated with schizophrenia. Among the 98 carriers of rs370429, 67 were affected by schizophrenia [111]. However, investigations in Japanese populations have not revealed any linkages between *pcm-1* and schizophrenia [112,113]. In animal experiments, *pcm-1*+/− mice demonstrate behavioral abnormalities, impairment in social interactions, and significantly reduced activity in the open field. However, mutant mice behave normally in the elevated plus maze, rotarod, prepulse inhibition, and progressive ratio tests [114].

## 4. Pericentrin (PCNT)

Pericentrin (PCNT) is also known as kendrin. This protein is constitutively localized in the microtubule-organizing center and is indispensable for the assembly of the pericentriolar matrix [59,60]. PCNT is localized in basal bodies and interacts with proteins involved in cilia assembly; *pcnt* silencing causes the inhibition of primary cilia formation [115]. An increased expression of *PCNT* in the postmortem brains and in the peripheral blood lymphocytes of bipolar disorder patients, when compared to healthy controls, has been demonstrated, although no SNP in *PCNT* associated with bipolar disorder were found [116]. However, the same team found significant allelic and genotypic associations of *PCNT* with schizophrenia in a Japanese population [117]. Differences in allelic frequencies or genotypic distributions of *PCNT* SNPs, between controls and schizophrenia patients, however, were not found in other studies [118]. Nevertheless, it was established that mutations in the *pcnt* lead to abnormal interneuron migration in the murine olfactory bulb, whereas schizophrenia is known to be accompanied by reduced olfactory bulb volume [61]. Pericentrin also interacts with Disc1 [119] and PCM-1 [55], which are essential for the keeping the central nervous system in a healthy condition, and in diseases. PCNT might also be important for susceptibility to dyslexia, because *PCNT* is localized on the chromosome region 21q22.3, and a deletion in this region was associated with dyslexia in the case of a dyslectic father and his three sons [120].

## 5. Abelson Helper Integration Site 1 (AHI1)

AHI1 (abelson helper integration site 1) is a cytoplasmic protein. AHI1 is associated with Joubert syndrome [121,122], otherwise known as jouberin. Mutations in *AHI1* are identified in 12% of patients with Joubert syndrome [123]. In a cell, AHI1 is localized in the transitional zone. It is involved in a protein complex, which serves as a barrier for non-ciliary-membrane proteins, preventing them from diffusing into the ciliary membrane [62].

The murine orthologue of AHI1 regulates cilia assembly via interaction with Rab8a: in mouse *ahi1*-knockdown cells, the ciliogenesis was impaired, and Rab8a was destabilized and did not properly localize to the basal body. Moreover, defects in the trafficking of endocytic vesicles from the plasma membrane to the Golgi complex and back to the plasma membrane were observed in *ahi1*-knockdown cells [63]. Interestingly, another cilia-associated protein PCM-1 is also involved in cilia formation via interaction with CEP290, whose complex CEP290−PCM-1 targets Rab8a in ciliogenesis [105]. It remains unknown whether PCM-1 and AHI1 work cooperatively in recruiting the Rab8a in ciliogenesis, or through different mechanisms.

So, as AHI1 is a pivotal protein for cilia formation and function, the knockdown of *ahi1* leads to the impairment of ciliogenesis. Reportedly, *ahi1* knockdown causes developmental abnormalities. For instance, in *ahi1* knockdown zebrafish, the loss of cilia in the Kupffer′s vesicle, and subsequent defects in cardiac left–right asymmetry, were demonstrated [64].

AHI1 interacts with β-catenin and facilitates its accumulation in the nucleus, positively modulating Wnt signaling [124]. In ciliated murine embryonic fibroblasts, the nuclear level of AHI1 and β-catenin is reduced in comparison to cells bearing primary cilia, i.e., nonmotile cilia disturb canonical Wnt signaling through a compartmentalization of its components. This repressive regulation does not silence the pathway, but maintains a discrete range of Wnt responsiveness; cells without cilia potentiate Wnt responses, whereas in cells with more than one cilium, responses are inhibited [125].

Mouse Ahi1 forms a stable complex with huntingtin-associated protein 1 (Hap1), which is involved in intracellular trafficking and is pivotal for neonatal development. The altered expression of *hap1* causes a reduced Ahi1 level, and vice versa, *ahi1* deficiency reduces the level of Hap1 [126]. Hap1 and Ahi1 stabilize each other, and are important for maintaining the level of tyrosine kinase receptor B (TrkB) [126], whose signaling seems to be critical in the risk of depression and bipolar disorder [65], and pivotal for brain development [126]. Interaction with HAP1 is also established for another cilia basal body associated protein, PCM-1, whose depletion leads to the impairment of primary cilia formation [106].

*AHI1* is expressed in the adult brain of both rodents and humans [126,127,128]. People with Joubert syndrome are characterized by abnormalities of the brainstem and cerebellum, weakness, clumsiness, and cognitive difficulties [121,129]. Brain polarity-associated disorders are shown to be associated with *AHI1* gene alteration. Potential evidence of the association between some variants of *AHI1* and bipolar disorder susceptibility has been reported, but no connections with clinical outcomes were revealed [65].

Connections between *AHI1* and schizophrenia vulnerability were established in several studies in various populations [130,131,132,133]. Moreover, a possible link between an *AHI1* SNP and a clinical outcome in patients with schizophrenia was found [134]. However, no differences in brain expression of *AHI1* in patients with schizophrenia or bipolar disorder, when compared to healthy people, were revealed [135].

## 6. Hamartin (Tuberous Sclerosis 1—TSC1)

The *TSC1* gene encodes hamartin. Mutations in this gene are associated with tuberous sclerosis, also known as tuberous sclerosis complex (TSC); hence the gene was named *tuberous sclerosis-1* and the protein name hamartin is from the hamartias, distinctive tumor-like malformations in a wide range of human tissues, characterizing the physical manifestation of this disease [136]. Patients with tuberous sclerosis often develop multiple tumors and it has been suggested that the *TSC1* is a tumor suppressor. Overexpression of *TSC1* leads to both cell growth inhibition and cell morphology changes [137]. The growth inhibition is associated with an increase in the endogenous level of tuberin, a product of the *TSC2* gene. A complex with hamartin-stabilized tuberin saved both proteins from ubiquitination, resulting in cell growth inhibition [137].

The hamartin-tuberin complex can also inhibit the mammalian target of rapamycin (mTOR) signaling, resulting in the inhibition of translational initiator S6 kinase 1 and of the inhibitor of translational initiation 4E binding protein 1 [138]. Acting as a GTPase-activating protein in the Rheb complex, hamartin-tuberin regulate mTOR; a lack of hamartin or tuberin causes an increase of Rheb-GTPs, which, in turn, causes a constitutive activation of mTOR signaling. This results in deregulation of the cell cycle and gene expression [139].

Hamartin is localized to the centrosome and can interact with the mitotic kinase Plk1. *tsc1*−/− murine embryonic fibroblasts show an increased number of centrosomes, when compared to *tsc1*+/+ cells [140]. Besides the centrosome, hamartin is localized in the basal body of the primary cilia. The loss of hamartin enhances ciliary formation: murine embryonic fibroblasts from *tsc1*−/− animals had a higher quantity of ciliated cells, than cells from control *tsc1*+/+ mice [68]. Disturbances in *tsc1* expression cause a difference in cilia length [68,141]. Furthermore, mice with a broken-down *tsc1* gene exhibited a significant reduction in dendritic spine density, in comparison with neuronal dendrites from control mice [69].

Two *tsc1* homologs, referred to as *tsc1a* and *tsc1b,* were found in zebrafish. In *tsc1a* knockdown fish, elongation of cilia, and defects in left-right visceral asymmetry, were observed. Moreover, kidney cyst formation in ciliary mutants was blocked by the TOR inhibitor, rapamycin [70].

Altogether, hamartin, or the hamartin−tuberin complex, interacts with more than 50 proteins. Besides tuberin, hamartin also forms complexes with proteins: DOCK7, ezrin/radixin/moesin, FIP200, IKKb, Melted, Merlin, NADE (p75NTR), NF-L, Plk1, and TBC7. It has not been shown whether the proteins interacting with hamartin also form complexes with tuberin, apart from Plk1 and TBC7, which are known not to interact with tuberin [71]. The hamartin−tuberin complex is involved in at least three signaling pathways: the PI3K-Akt pathway, the ERK1/2-RSK1 pathway, and the LKB1-AMPK pathway. So, hamartin-tuberin is recruited in the cell cycle, metabolism, and cell polarity control. Acting in the brain, the hamartin-tuberin complex is involved in neuronal arborization, which constitutes the regulation of spine density as a part of the PI3K-Akt-mTOR pathway [142].

A characteristic feature of patients with tuberous sclerosis is autism [143,144,145]. Heterozygous or homozygous loss of *tsc1* in murine cerebellar Purkinje cells, leads to autistic-like behaviors, including abnormal social interaction, repetitive behavior, and vocalizations, while treatment with rapamycin abolishes this misbehavior [146]. The hamartin-tuberin complex also negatively regulates β-catenin stability and activity, by participating in β-catenin degradation complex [147]. It is suggested that the activity of the canonical Wnt pathway is altered, at least in a subset of patients, with autism spectrum disorder [148].

## 7. DCDC2

While its function remains undisclosed, DCDC2 has been shown to be associated with dyslexia [149,150]. *DCDC2* is expressed in the fetal and adult human CNS [150]. It is localized to the brain regions which are active at the time of cursory reading. DCDC2 belongs to the doublecortin family, which is characterized by an ability to bind microtubules and by an involvement in neuronal migration [72]. *dcdc2* silencing in rat embryos results in an impairment of neuronal migration [149]. The DCDC2 protein is localized to the primary cilium axoneme and its overexpression results in cilia length enhancement [72,151]. When *dcdc2* is overexpressed in rat hippocampal cells, an aberrant morphology of neurite outgrowth is observed: the branching of neurites increases, although their total length does not change significantly [72]. A knockdown of *dcdc2* disrupts ciliogenesis in a murine kidney cell line IMCD-3, but ciliogenesis in affected cells may be rescued by artificially-induced wild-type human *DCDC2* expression [151]. Animal studies revealed that *dcdc2* mutations cause a renal-hepatic ciliopathy in murine models and lead to ciliopathy phenotypes in zebrafish [151]. *dcdc2* zebrafish orthologue is also involved in left-right patterning [151].

DCDC2 is involved in ciliary signaling: an overexpression of *dcdc2* triggers Shh signaling, whereas *dcdc2* downregulation by shRNA, leads to Wnt signaling [72,151]. DCDC2 interacts with disheveled proteins 1−3, while *DCDC2* overexpression represses β-catenin-dependent Wnt signaling [151]. Zebrafish ciliopathy phenotype in *dcdc2* morphants can be rescued by the addition of a β-catenin inhibitor [151]. In addition, DCDC2 is localized in the kinocilia of sensory hair cells and the primary cilia of nonsensory supporting cells. A missense mutation in *DCDC2* caused deafness in a Tunisian family [152]. Although *DCDC2* was described as a susceptibility gene for dyslexia in the United States [149], Germany [150], and Italy [153], studies in a UK population have only revealed weak, inconsistent evidence for *DCDC2* involvement in dyslexia [154]. Moreover, a linkage between *DCDC2* SNPs and gray matter volumes in the superior prefrontal, temporal, and occipital networks (regions including multiple reading and language-related areas), has been found; linkages were observed in subjects with schizophrenia, but not in the control group [155]. An association between the SNPs rs793842 and rs3743204 (in *DCDC2* and *dyx1c1* genes respectively), and the volume of the white matter in the left temporo-parietal region, was also shown. Although an association between the white matter volume and reading scores was found, these SNPs did not appear to be linked with reading skills [156].

Considering that mutations in doublecortin genes lead to neuronal migration impairment, and RNA knockdown disturbs neuron progenitors migration in rat embryos [149], it has been suggested that defects in the *DCDC2* gene cause dyslexia by means of incorrect migration of neural progenitor cells.

## 8. DYX1C1

The Dyx1c1 gene is involved in dyslexia; its expression in a set of cortical neurons and glial cells occurs in white matter [157]. Based on a large set of published microarray data, it has been suggested that Dyx1c1 belongs to ciliary proteins [158]. In the primary cilia of some cells, the Dyx1c1-GFP fusion protein was co-localized with γ-tubulin at the centrosome, and in some other cells, in a primary cilia axoneme [159].

As shown by the use of mRNA in in situ hybridization, *dyx1c1* is expressed in many ciliated tissues in zebrafish, both adult and fetal. In *dyx1c1* morphants, cilia length is reduced in several organs, including the Kupffer’s vesicle [73]. Furthermore, the loss of both outer and inner dynein arms, which are required for cilia motility, was detected in *dyx1c1* morphants [73]. The defects in dynein arms were found in primary ciliary dyskinesia patients bearing a mutation in *dyx1c1* [74]. A loss-of-function of *dyx1c1* in zebrafish also causes aberrations in organ asymmetry: heart looping, coiling of the gut, position of the liver, and the breakdown of the pancreas. Additionally, the position of the parapineal organ was reversed in a part of *dyx1c1* morphant embryos [73,74]. Moreover, Tarkar and colleages found the situs inversus phenotype in five out of 12 primary ciliary dyskinesia patients with recessive mutations in *dyx1c1*, and two individuals had aberrations in left-right asymmetry (one with dextrocardia and polysplenia, and one with left atrial isomerism and polysplenia) [74].

The association between some *Dyx1c1* SNPs and dyslexia has been recorded in various populations [160,161,162]. Animal studies have shown that mice with the homozygous *Dyx1c1* knockout, demonstrate memory and learning deficits [163]. In utero knockdown of *Dyx1c1*, disrupted neuronal migration has been recorded in the developing neocortex of rat embryos [75,76]. Neuronal migration abnormalities were also observed in the brains of dyslexic patients [76].

## 9. General Discussion

When starting this review, we expected to demonstrate some linkage between ciliary proteins and neurodevelopmental disorders, based on an involvement of primary cilia in the development of brain asymmetry. However, we failed to find any evidence to confirm this hypothesis. Although these proteins are associated with primary cilia, they affect neurodevelopmental disorders by other mechanisms. The way in which aberrations in gene expression lead to behavior impairment, is still not entirely clear. There are several ways in which the proteins reviewed here can influence the development of mental disorders: by affecting neuronal migration, by influencing neurites outgrowth, or by alternating cellular and interneuron signaling (Table 1).

Alterations in the expression of the ciliary proteins lead to incorrect neuronal migration (Table 1), which is a crucial event for the formation of the cerebral cortex. Mental retardation or cognitive defects are observed in some ciliopathies [164,165], suggesting that cilia are important for brain development and functioning. Aberrations of neuronal precursor cell migration are known in schizophrenia [166], autism [167], bipolar disorder [168], and dyslexia [169], although there is also conflicting evidence for this [170]. For example, incorrect neuronal migration, caused by a *Disc1* single-nucleotide polymorphism, leads to a deficiency in the grey matter volume in some brain areas in patients with major depressive disorders [80], while that caused by polymorphisms in *dyx1c1* or *dcdc2*, leads to changes in temporo-parietal white matter structure [156]. Disc1, PCM-1, PCNT, and TSC1 are located, and have functions in, the centrosome (Table 1). This organelle, as the main microtubular cytoskeleton organizing center, is implicated in various processes during the development of the nervous system, particularly in neuronal migration and polarization [171]. Thus, mutations in the genes coding the proteins under review result in incorrect functioning of the centrosome. This may, in turn, lead to neurodevelopmental disorders, probably via incorrect neuronal precursor migration.

Apart from neuronal migration, centrosomes are involved in neurite growth and branching. Its incorrect functioning, therefore, reflects breakages in interrelations of neurons by disturbance of neuritis and incorrect spindle orientation [172]. In a recent study, the dendrite arborization was shown to depend on normal ciliogenesis: disruption of normal ciliogenesis impaired dendrite outgrowth [173]. An alteration of the expression of ciliary protein genes causes a disturbance of the neurite net. Disc1 is involved in the neurite outgrowth [89]. Other proteins are also implicated in the neuronal net construction: abberations in the expression of *disc1* and *tsc1* causes a decrease in dendritic spines [69,174], while an overexpression of *dcdc2* leads to an increase in neurite branching [72], and the expression of truncated *ahi1* results in inhibition of neurite outgrowth [126]. Breakages in the function of cilia genes lead to aberrant brain development, which causes functional and structural brain abnormalities, observed, e.g., in schizophrenic [175,176], autistic [177], and dyslexic [156] patients.

Furthermore, Disc1 is recruited in GABA_A_R-trafficking in cortical neurons, via its involvement in microtubule-associated cellular transport: its knockdown or overexpression alters distribution of GABA_A_R on the neuron′s surface [96]. Mutations in *disc1* lead to the reduction of released GABA, due to alterations of cellular traffic [51]. Disc1 regulates dendrite development during adult neurogenesis and this regulation requires GABA-induced depolarization, through a convergence with the AKT-mTOR pathway [95]. Recruitment of mTOR signaling leads to speculation that TSC1 is involved in GABA signaling. Indeed, in conditional knockout mice with deletion of the *tsc1* gene in GABAergic interneuron progenitor cells, the number of GABAergic neurons in the cortex and the dentate gyrus, was decreased; moreover, the upregulation of mTORC1 signaling and increased GABAergic interneuron size were observed [178]. Hap1, the binding partner of PCM-1, TSC1, and AHI1, is implicated in the rapid delivery of GABA_A_Rs to inhibitory synapses [179]. Also, *dyx1c1* knockdown in utero causes a non-cell autonomous effect on GABAergic neuronal migration [180]. So, GABA is another way by which defects in ciliary proteins can provoke mental disorders. GABA is the key inhibitory neurotransmitter involved in hyperpolarization of the neurons. It is also an important mediator in the regulation of adult neurogenesis, being involved in proliferation, migration, and synaptic integration of the neurons [181]. Aberrations of the GABA-pathway are associated with neural disorders, such as schizophrenia [182,183], bipolar disorder [184], and autism [185].

Primary cilia are implicated in some paracrine signaling pathways. In particular, cilia play crucial roles in hedgehog and wnt signaling [1], i.e., in signaling pathways that take part in neurogenesis [58,186,187], and whose failures are associated with neurodevelopmental diseases [148,188]. For the majority of the considered proteins, a direct involvement in these signaling pathways has been shown (Table 1).

**Figure 2:**
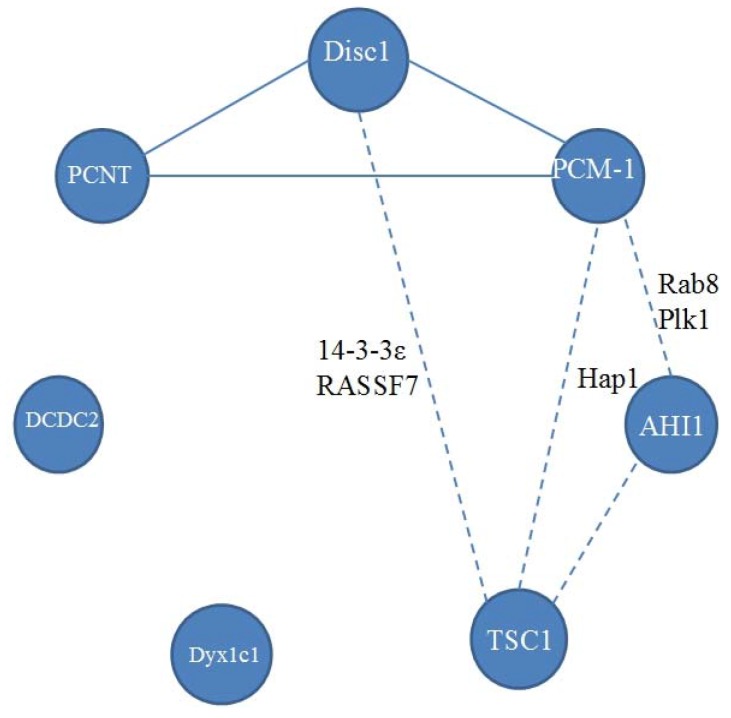
A scheme of known relations between proteins under review. Continuous line between proteins means direct interactions. Dashed line means that a pair of proteins has common binding partners, their names are given near the dashed line.

**Figure 3:**
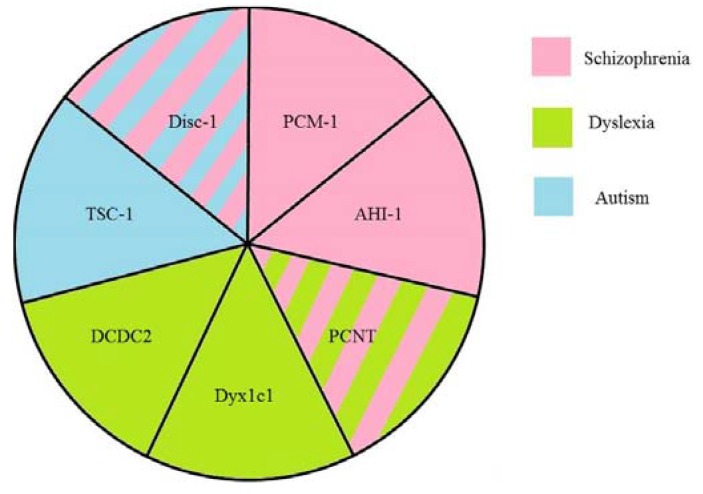
A scheme of suggested associations between the ciliary proteins and neurodevelopmental diseases. Striped sectors indicate that the protein is associated with at least two diseases. Note different location in the cilia or protein complexes of those ciliary proteins related to different diseases.

The known relations between reviewed proteins are presented in Figure 2, and their associations to neurodevelopmental disease are schematized in Figure 3. Disc1 can contact PCM-1 and PCNT. Each of these three proteins has been shown to be associated with schizophrenia. It is unknown, however, whether PCNT, Disc1, and PCM-1, act independently or in an orchestrated manner in neurodevelopmental disorders. Disturbance in this complex may affect cell division in the central nervous system. This proposition is supported by the finding that PCM-1-associated schizophrenia patients have orbitofrontal volumetric deficits [110]. PCM-1 and AHI1 can interact with Rab8 and Plk1. However, although PCM-1 and AHI1 are associated with schizophrenia, there are no observations of implication of either Plk1 or Rab8, into pathogenesis of schizophrenia.

TSC1 and Disc1 interact with 14-3-3 proteins. 14-3-3 proteins are a family of regulatory proteins, involved in cell cycle regulation and apoptosis [189]. They express during development and in adulthood. There is evidence that disturbances in the quantities of these proteins in the cell, are implicated in neurological disorders with polarity breakdown: autism [190,191,192], bipolar disorder [193,194], and schizophrenia [195,196]. Interestingly, 14-3-3 proteins are recruited in vertebrate left-right axis determination [197]. The TSC1-TSC2 complex is capable of interacting with all proteins from the 14-3-3 family. There is no evidence of direct interaction of TSC1 with 14-3-3 proteins, but it is likely that the interaction is carried out through TSC2. The binding of the 14-3-3 protein to the TSC1-TSC2 complex prevents an inhibition of S6 kinase and the non-phosphorylation of 4E binding protein 1, i.e., it causes a weakening of the PI3K/PKB/TOR pathway signaling [198]. Conversely, cellular levels of some 14-3-3 proteins (γ, ε, σ, and ζ) are regulated by TSC1 and TSC2 [199]. Disc1 can also interact with 14-3-3 proteins, in particular, with 14-3-3ζ [200] and 14-3-3ε [201]. While 14-3-3 proteins and Disc-1 are known to be associated with schizophrenia, no such evidence exists for TSC1.

Another common partner of Disc-1 and hamartin, is RASSF7, a member of a family of Ras association domain–containing proteins (RASSF). It is a centrosomal protein. Its knockdown in *Xenopus* causes nuclear breakdown, apoptosis, and a striking loss of tissue architecture in the neural tube [202], but its associations with neurodevelopmental disorders, with which Disc-1 or TSC1 have linkages, are still unknown. Futhermore, no connections between DCDC2 and Dyx1c1, or with other ciliary proteins, have been revealed.

On the one hand, the majority of the considered ciliary proteins affect visceral left-right asymmetry (Table 1), whereas mental disorders are associated with failures in lateralization. On the other hand, there is no direct evidence that these proteins are involved in neuropathological processes through the mechanisms underlying visceral asymmetry. Therefore, a divergence in developmental mechanisms underlying the establishment of visceral and brain asymmetries can be suggested [27,28,29]. This view is supported by a recent finding by Vingerhoets and colleagues [203], that only situs inversus totalis patients with ciliopathies preserve sidedness of the neurobehahavioural phenotypes. In contrast, those patients with situs inversus totalis, who express no symptoms of ciliopathies, display a tendency to randomize neurobehavioural asymmetries, suggesting that two different developmental mechanisms underlie visceral situs. All these data explain why ciliary proteins may be associated with both mental disorders and visceral asymmetries, but the patients may not necessarily express both psychiatric and inverted visceral phenotypes. They also indicate why mental disorders and visceral inversions may be related to ciliary (no doubt, various) malfunctions, but not necessarily to left-right neurobehavioural asymmetries, at the same time [203]. Since disturbances in ciliary genes lead to mental disorders, we can expect that findings of other ciliary genes, specifically of their mutations, will indicate that they may lead to psychiatry phenotypes.

## Figures and Tables

**Table 1:** Ciliary protein functions, involved in visceral asymmetry and neurodevelopmental pathogenesis.

Protein	Function in the Cilia	Other Functions	Involvement in Visceral Asymmetry	Associated Psychiatric Disorders	Suggested Mechanisms	Ref.
Disc1	ciliogenesis and intraflagellar transport regulation	microtubular transport, probably mitochondrial protein import machinery, Akt/mTOR and GSK-3/β-catenin/Wnt pathways		schizophrenia, autism, depression, bipolar disorder	neuronal migration, neuronal signaling and signal transduction, axonal bundling, transport of GABA-containing vesicles	[49,50,51,52,53,54]
PCM-1	ciliogenesis and cilia disassembly	microtubule-based trafficking of proteins to the centrosome, centrosome assembly	heart left-right asymmetry in zebrafish	schizophrenia	cell cycle regulation and migration of neurons alone or in coordination with Disc1	[55,56,57,58]
PCNT	interacts with proteins involved in cilia assembly	pericentriolar matrix assembly, anchors the γ-tubulin complex to the centrosome, providing microtubule nucleation sites		dyslexia schizophrenia	functioning of the centrosomes and the cytoskeleton, interneuron migration	[59,60,61]
AHI1	prevention of non-ciliarymembrane proteins from diffusing into the ciliary membrane, cilia assembly via interaction with Rab8a	traffic of endocytic vesicles	heart looping in zebrafish knockdown	schizophrenia bipolar disorder	in complex with Hap1 maintains the level of TrkB, neuronal migration	[62,63,64,65,66,67]
TSC1	inhibits formation of the extra cilia	cell cycle regulation, methabolism, cell polarity, mTOR, PI3K-Akt, the ERK1/2-RSK1 signaling	affected expression of *southpaw* gene in zebrafish morphants	autism	maintenance of dendrite spine density, mTOR signaling pathway, neuronal migration	[68,69,70,71]
DCDC2	ciliogenesis and ciliary signaling	promotes Shh signaling and inhibits Wnt signaling	left-right asymmetry defects in liver, gut, and pancreas	dyslexia	maintenance of the balance between Shh and Wnt signaling, neuronal migration	[13,42,72]
DYX1C1	ciliogenesis and cilia motility (dynein arm assembly)		normal heart looping, left-right asymmetry defects in liver, gut, and pancreas	dyslexia	neuronal migration	[73,74,75,76]

## References

[1] Singla V., Reiter J.F. (2006). The primary cilium as the cell’s antenna: Signaling at a sensory organelle. Science.

[2] Del Bigio M.R. (1995). The ependyma: A protective barrier between brain and cerebrospinal fluid. Glia.

[3] Sawamoto K., Wichterle H., Gonzalez-Perez O., Cholfin J.A., Yamada M., Spassky N., Murcia N.S., Garcia-Verdugo J.M., Marin O., Rubenstein J.L. (2006). New neurons follow the flow of cerebrospinal fluid in the adult brain. Science.

[4] Ibanez-Tallon I., Pagenstecher A., Fliegauf M., Olbrich H., Kispert A., Ketelsen U.P., North A., Heintz N., Omran H. (2004). Dysfunction of axonemal dynein heavy chain Mdnah5 inhibits ependymal flow and reveals a novel mechanism for hydrocephalus formation. Hum. Mol. Genet..

[5] Thomson P.A., Malavasi E.L., Grunewald E., Soares D.C., Borkowska M., Millar J.K. (2013). DISC1 genetics, biology and psychiatric illness. Front. Biol..

[6] Lee J.E., Gleeson J.G. (2011). Cilia in the nervous system: Linking cilia function and neurodevelopmental disorders. Curr. Opin. Neurol..

[7] Davis E.E., Katsanis N. (2012). The ciliopathies: A transitional model into systems biology of human genetic disease. Curr. Opin. Gen. Dev..

[8] Afzelius B.A. (1976). A human syndrome caused by immotile cilia. Science.

[9] Rott H.D. (1979). Kartagener′s syndrome and the syndrome of immotile cilia. Hum. Genet..

[10] Nonaka S., Shiratori H., Saijoh Y., Hamada H. (2002). Determination of left-right patterning of the mouse embryo by artificial nodal flow. Nature.

[11] Nonaka S., Tanaka Y., Okada Y., Takeda S., Harada A., Kanai Y., Kido M., Hirokawa N. (1998). Randomization of left-right asymmetry due to loss of nodal cilia generating leftward flow of extraembryonic fluid in mice lacking KIF3B motor protein. Cell.

[12] Kamura K., Kobayashi D., Uehara Y., Koshida S., Iijima N., Kudo A., Yokoyama T., Takeda H. (2011). Pkd1l1 complexes with Pkd2 on motile cilia and functions to establish the left-right axis. Development.

[13] McGrath J., Somlo S., Makova S., Tian X., Brueckner M. (2003). Two populations of node monocilia initiate left-right asymmetry in the mouse. Cell.

[14] Babu D., Roy S. (2013). Left-right asymmetry: cilia stir up new surprises in the node. Open Biol..

[15] Essner J.J., Vogan K.J., Wagner M.K., Tabin C.J., Yost H.J., Brueckner M. (2002). Conserved function for embryonic nodal cilia. Nature.

[16] Essner J.J., Amack J.D., Nyholm M.K., Harris E.B., Yost H.J. (2005). Kupffer′s vesicle is a ciliated organ of asymmetry in the zebrafish embryo that initiates left-right development of the brain, heart and gut. Development.

[17] Schweickert A., Weber T., Beyer T., Vick P., Bogusch S., Feistel K., Blum M. (2007). Cilia-driven leftward flow determines laterality in Xenopus. Curr. Biol..

[18] Dathe V., Gamel A., Manner J., Brand-Saberi B., Christ B. (2002). Morphological left-right asymmetry of Hensen’s node precedes the asymmetric expression of *Shh.* and *Fgf-8* in the chick embryo. Anat. Embryol..

[19] Nishide K., Mugitani M., Kumano G., Nishida H. (2012). Neurula rotation determines left-right asymmetry in ascidian tadpole larvae. Development.

[20] Concha M.L., Burdine R.D., Russell C., Schier A.F., Wilson S.W. (2000). A Nodal signaling pathway regulates the laterality of neuroanatomical asymmetries in the zebrafish forebrain. Neuron.

[21] Roussigne M., Bianco I.H., Wilson S.W., Blader P. (2009). Nodal signaling imposes left-right asymmetry upon neurogenesis in the habenular nuclei. Development.

[22] Barth K.A., Miklosi A., Watkins J., Bianco I.H., Wilson S.W., Andrew R.J. (2005). *Fsi.* zebrafish show concordant reversal of laterality of viscera, neuroanatomy, and a subset of behavioral responses. Curr. Biol..

[23] Roussigne M., Blader P., Wilson S.W. (2012). Breaking symmetry: The zebrafish as a model for understanding left-right asymmetry in the developing brain. Dev. Neurobiol..

[24] Kennedy D.N., O′Craven K.M., Ticho B.S., Goldstein A.M., Makris N., Henson J.W. (1999). Structural and functional brain asymmetries in human situs inversus totalis. Neurology.

[25] McManus C. (2005). Reversed bodies, reversed brains, and (some) reversed behaviors: of zebrafish and men. Dev. Cell..

[26] Tanaka S., Kanzaki R., Yoshibayashi M., Kamiya T., Sugishita M. (1999). Dichotic listening in patients with situs inversus: Brain asymmetry and situs asymmetry. Neuropsychologia.

[27] Ihara A., Hirata M., Fujimaki N., Goto T., Umekawa Y., Fujita N., Terazono Y., Matani A., Wei Q., Yoshimine T. (2010). Neuroimaging study on brain asymmetries in situs inversus totalis. J. Neurol. Sci..

[28] Malashichev Y., Malashichev Y.B., Deckel W. (2006). Is there a link between visceral and neurobehavioral asymmetries in development and evolution?. Behavioral and Morphological Asymmetries in Veretbrates. Georgetown.

[29] Malashichev Y.B., Wassersug R.J. (2004). Left and right in the amphibian world: Which way to develop and where to turn?. BioEssays.

[30] Trulioff A.S., Malashichev Y.B., Ermakov A.S. (2015). Artificial inversion of the left-right visceral asymmetry in vertebrates: Conceptual approaches and experimental solutions. Russ. J. Dev. Biol..

[31] Clarke J.M., Zaidel E. (1994). Anatomical-behavioral relationships: Corpus callosum morphometry and hemispheric specialization. Behav. Brain Res..

[32] Witelson S.F., Nowakowski R.S. (1991). Left out axons make men right: A hypothesis for the origin of handedness and functional asymmetry. Neuropsychologia.

[33] Brandler W.M., Morris A.P., Evans D.M., Scerri T.S., Kemp J.P., Timpson N.J., St Pourcain B., Smith G.D., Ring S.M., Stein J. (2013). Common variants in left/right asymmetry genes and pathways are associated with relative hand skill. PLoS Genet..

[34] Halpern M.E., Gunturkun O., Hopkins W.D., Rogers L.J. (2005). Lateralization of the vertebrate brain: Taking the side of model systems. J. Neurosci..

[35] Rogers L.J. (2014). Asymmetry of brain and behavior in animals: Its development, function, and human relevance. Genesis.

[36] Herbert M.R., Ziegler D.A., Deutsch C.K., O′Brien L.M., Kennedy D.N., Filipek P.A. (2005). Brain asymmetries in autism and developmental language disorder: A nested whole-brain analysis. Brain.

[37] Robichon F., Levrier O., Farnarier P., Habib M. (2000). Developmental dyslexia: Atypical cortical asymmetries and functional significance. Eur. J. Neurol..

[38] Pujol J., Cardoner N., Benlloch L., Urretavizcaya M., Deus J., Losilla J.M., Bakardjiev A.I., Hodgson J., Takeoka M., Makris N. (2002). CSF spaces of the Sylvian fissure region in severe melancholic depression. Neuroimage.

[39] Crow T.J., Done D.J., Sacker A. (1996). Cerebral lateralization is delayed in children who later develop schizophrenia. Schizophr. Res..

[40] Petty R.G. (1999). Structural asymmetries of the human brain and their disturbance in schizophrenia. Schizophr. Bull..

[41] Finkelstein B.A. (1962). Mental symptoms occurring in Kartagener′s syndrome. Am. J. Psychiatry.

[42] Glick I.D., Graubert D.N. (1964). Kartagener′s syndrome and schizophrenia: A report of a case with chromosomal studies. Am. J. Psychiatry.

[43] Mohan I., Lowe M., Sundram S. (2013). Comorbid situs inversus totalis and schizophrenia in a young male. Aust. N. Z. J. Psychiatry.

[44] Quast T.M., Sippert J.D., Sauve W.M., Deutsch S.I. (2005). Comorbid presentation of Kartagener′s syndrome and schizophrenia: Support of an etiologic hypothesis of anomalous development of cerebral asymmetry?. Schizophr. Res..

[45] Ermiş A., Turkcan A., Ceylan M.E., Maner A.F. (2009). Kartagener Sendromu ve Psikotik Bozukluk: Olgu Sunumu. J. Psychiatry Neurol. Sci..

[46] Kondziella D., Lycke J. (2008). Autism spectrum disorders: does cilia dysfunction in embryogenesis play a role?. Acta Neuropsychiatr..

[47] Suzuki T., Washio Y., Aritaki M., Fujinami Y., Shimizu D., Uji S., Hashimoto H. (2009). Metamorphic *pitx2* expression in the left habenula correlated with lateralization of eye-sidedness in flounder. Dev. Growth Differ..

[48] Compagnucci C., Fish J., Depew M.J. (2014). Left-right asymmetry of the gnathostome skull: Its evolutionary, developmental, and functional aspects. Genesis.

[49] Marley A., von Zastrow M. (2010). DISC1 regulates primary cilia that display specific dopamine receptors. PLoS ONE.

[50] Morris J.A., Kandpal G., Ma L., Austin C.P. (2003). DISC1 (Disrupted-In-Schizophrenia 1) is a centrosome associated protein that interacts with MAP1A, MIPT3, ATF4/5 and NUDEL: Regulation and loss of interaction with mutation. Hum. Mol. Genet..

[51] Berridge M.J. (2014). Calcium signalling and psychiatric disease: Bipolar disorder and schizophrenia. Cell Tissue Res..

[52] James R., Adams R.R., Christie S., Buchanan S.R., Porteous D.J., Millar J.K. (2004). Disrupted in Schizophrenia 1 (DISC1) is a multicompartmentalized protein that predominantly localizes to mitochondria. Mol. Cell. Neurosci..

[53] Park Y.U., Jeong J., Lee H., Mun J.Y., Kim J.H., Lee J.S., Nguyen M.D., Han S.S., Suh P.G., Park S.K. (2010). Disrupted-in-schizophrenia 1 (DISC1) plays essential roles in mitochondria in collaboration with Mitofilin. Proc. Natl. Acad. Sci. USA.

[54] Balu D.T., Coyle J.T. (2011). Neuroplasticity signaling pathways linked to the pathophysiology of schizophrenia. Neurosci. Biobehav. Rev..

[55] Dammermann A., Merdes A. (2002). Assembly of centrosomal proteins and microtubule organization depends on PCM-1. J. Cell Biol..

[56] Kamiya A., Tan P.L., Kubo K.I., Engelhard C., Ishizuka K., Kubo A., Tsukita S., Pulver A.E., Nakajima K., Cascella N.G. (2008). Recruitment of PCM1 to the centrosome by the cooperative action of DISC1 and BBS4: A candidate for psychiatric illnesses. Arch. Gen. Psychiatry.

[57] Wang G., Chen Q., Zhang X., Zhang B., Zhuo X., Liu J., Jiang Q., Zhang C. (2013). PCM1 recruits Plk1 to the pericentriolar matrix to promote primary cilia disassembly before mitotic entry. J. Cell Sci..

[58] Joksimovic M., Yun B.A., Kittappa R., Anderegg A.M., Chang W.W., Taketo M.M., McKay R.D., Awatramani R.B. (2009). Wnt antagonism of Shh facilitates midbrain floor plate neurogenesis. Nature Neurosci..

[59] Doxsey S.J., Stein P., Evans L., Calarco P.D., Kirschner M. (1994). Pericentrin, a highly conserved centrosome protein involved in microtubule organization. Cell.

[60] Takahashi M., Yamagiwa A., Nishimura T., Mukai H., Ono Y. (2002). Centrosomal proteins CG-NAP and kendrin provide microtubule nucleation sites by anchoring γ-tubulin ring complex. Mol. Biol. Cell.

[61] Endoh-Yamagami S., Karkar K.M., May S.R., Cobos I., Thwin M.T., Long J.E., Ashique A.M., Zarbalis K., Rubenstein J.L., Peterson A.S. (2010). A mutation in the pericentrin gene causes abnormal interneuron migration to the olfactory bulb in mice. Dev. Biol..

[62] Chih B., Liu P., Chinn Y., Chalouni C., Komuves L.G., Hass P.E., Sandoval W., Peterson A.S. (2012). A ciliopathy complex at the transition zone protects the cilia as a privileged membrane domain. Nature Cell Biol..

[63] Hsiao Y.-C., Tong Z.J., Westfall J.E., Ault J.G., Page-McCaw P.S., Ferland R.J. (2009). Ahi1, whose human ortholog is mutated in Joubert syndrome, is required for Rab8a localization, ciliogenesis and vesicle trafficking. Hum. Mol. Genet..

[64] Simms R.J., Hynes A.M., Eley L., Inglis D., Chaudhry B., Dawe H.R., Sayer J.A. (2012). Modelling a ciliopathy: Ahi1 knockdown in model systems reveals an essential role in brain, retinal, and renal development. Cell. Mol. Life Sci..

[65] Porcelli S., Pae C.-U., Han C., Lee S.-J., Patkar A.A., Masand P.S., Balzarro B., Alberti S., De Ronchi D., Serretti A. (2014). Abelson helper integration site-1 gene variants on major depressive disorder and bipolar disorder. Psychiatry Invest..

[66] Guo J., Higginbotham H., Li J., Nichols J., Hirt J., Ghukasyan V., Anton E.S. (2015). Developmental disruptions underlying brain abnormalities in ciliopathies. Nat. Commun..

[67] Torri F., Akelai A., Lupoli S., Sironi M., Amann-Zalcenstein D., Fumagalli M., Dal Fiume C., Ben-Asher E., Kanyas K., Cagliani R. (2010). Fine mapping of AHI1 as a schizophrenia susceptibility gene: From association to evolutionary evidence. FASEB J..

[68] Hartman T.R., Liu D., Zilfou J.T., Robb V., Morrison T., Watnick T., Henske E.P. (2009). The tuberous sclerosis proteins regulate formation of the primary cilium via a rapamycin-insensitive and polycystin 1—Independent pathway. Hum. Mol. Genet..

[69] Meikle L., Pollizzi K., Egnor A., Kramvis I., Lane H., Sahin M., Kwiatkowski D.J. (2008). Response of a neuronal model of tuberous sclerosis to mTOR inhibitors: effects on mTORC1 and Akt signaling lead to improved survival and function. J. Neurosci..

[70] DiBella L.M., Park A., Sun Z. (2009). Zebrafish Tsc1 reveals functional interactions between the cilium and the TOR pathway. Hum. Mol. Genet..

[71] Rosner M., Hanneder M., Siegel N., Valli A., Hengstschlager M. (2008). The tuberous sclerosis gene products hamartin and tuberin are multifunctional proteins with a wide spectrum of interacting partners. Mutat. Res..

[72] Massinen S., Hokkanen M.-E., Matsson H., Tammimies K., Tapia-Paez I., Dahlstrom-Heuser V., Kuja-Panula J., Burghoorn J., Jeppsson K.E., Swoboda P. (2011). Increased expression of the dyslexia candidate gene DCDC2 affects length and signaling of primary cilia in neurons. PLoS ONE.

[73] Chandrasekar G., Vesterlund L., Hultenby K., Tapia-Paez I., Kere J. (2013). The Zebrafish Orthologue of the Dyslexia Candidate Gene DYX1C1 Is Essential for Cilia Growth and Function. PLoS ONE.

[74] Tarkar A., Loges N.T., Slagle C.E., Francis R., Dougherty G.W., Tamayo J.V., Shook B., Cantino M., Schwartz D., Jahnke C. (2013). DYX1C1 is required for axonemal dynein assembly and ciliary motility. Nature Genet..

[75] Wang Y., Paramasivam M., Thomas A., Bai J., Kaminen-Ahola N., Kere J., Voskuil J., Rosen G.D., Galaburda A.M., Loturco J.J. (2006). DYX1C1 functions in neuronal migration in developing neocortex. Neuroscience.

[76] Rosen G.D., Bai J., Wang Y., Fiondella C.G., Threlkeld S.W., LoTurco J.J., Galaburda A.M. (2007). Disruption of neuronal migration by RNAi of Dyx1c1 results in neocortical and hippocampal malformations. Cereb. Cortex.

[77] St Clair D., Blackwood D., Muir W., Walker M., Carothers A., Spowart G., Gosden C., Evans H.J. (1990). Association within a family of a balanced autosomal translocation with major mental illness. Lancet.

[78] Millar J.K., Wilson-Annan J.C., Anderson S., Christie S., Taylor M.S., Semple C.A., Devon R.S., St Clair D.M., Muir W.J., Blackwood D.H. (2000). Disruption of two novel genes by a translocation co-segregating with schizophrenia. Hum. Mol. Genetics.

[79] Kilpinen H., Ylisaukko-Oja T., Hennah W., Palo O.M., Varilo T., Vanhala R., Nieminen-von Wendt T., Von Wendt L., Paunio T., Peltonen L. (2008). Association of DISC1 with autism and Asperger syndrome. Mol. Psychiatry.

[80] Hashimoto R., Numakawa T., Ohnishi T., Kumamaru E., Yagasaki Y., Ishimoto T., Mori T., Nemoto K., Adachi N., Izumi A. (2006). Impact of the DISC1 Ser704Cys polymorphism on risk for major depression, brain morphology and ERK signaling. Hum. Mol. Genet..

[81] Thomson P.A., Macintyre D.J., Hamilton G., Dominiczak A., Smith B.H., Morris A., Evans K.L., Porteous D.J. (2013). Association of DISC1 variants with age of onset in a population-based sample of recurrent major depression. Mol. Psychiatry.

[82] Hodgkinson C.A., Goldman D., Jaeger J., Persaud S., Kane J.M., Lipsky R.H., Malhotra A.K. (2004). Disrupted in schizophrenia 1 (DISC1): Association with schizophrenia, schizoaffective disorder, and bipolar disorder. Am. J. Hum. Genet..

[83] Hennah W., Thomson P., McQuillin A., Bass N., Loukola A., Anjorin A., Blackwood D., Curtis D., Deary I.J., Harris S.E. (2009). DISC1 association, heterogeneity and interplay in schizophrenia and bipolar disorder. Mol. Psychiatry.

[84] Maeda K., Nwulia E., Chang J., Balkissoon R., Ishizuka K., Chen H., Zandi P., McInnis M.G., Sawa A. (2006). Differential expression of disrupted-in-schizophrenia (DISC1) in bipolar disorder. Biol. Psychiatry.

[85] Li C., Inglis P.N., Leitch C.C., Efimenko E., Zaghloul N.A., Mok C.A., Davis E.E., Bialas N.J., Healey M.P., Héon E. (2008). An essential role for DYF-11/MIP-T3 in assembling functional intraflagellar transport complexes. PLoS Genet..

[86] Wang Q., Brandon N.J. (2011). Regulation of the cytoskeleton by Disrupted-in-Schizophrenia 1 (DISC1). Mol. Cell. Neurosci..

[87] Higginbotham H.R., Gleeson J.G. (2007). The centrosome in neuronal development. Trends Neurosci..

[88] Kam A., Kubo K.I., Tomoda T., Takaki M., Youn R., Ozeki Y., Sawamura N., Park U., Kudo C., Okawa M. (2005). A schizophrenia-associated mutation of DISC1 perturbs cerebral cortex development. Nat. Cell Biol..

[89] Kamiya A., Tomoda T., Chang J., Takaki M., Zhan C., Morita M., Cascio M.B., Elashvili S., Koizumi H., Takanezawa Y. (2006). DISC1-NDEL1/NUDEL protein interaction, an essential component for neurite outgrowth, is modulated by genetic variations of DISC1. Hum. Mol. Genet..

[90] Wang Q., Charych E.I., Pulito V.L., Lee J.B., Graziane N.M., Crozier R.A., Revilla-Sanchez R., Kelly M.P., Dunlop A.J., Murdoch H. (2011). The psychiatric disease risk factors DISC1 and TNIK interact to regulate synapse composition and function. Mol. Psychiatry.

[91] Mao Y., Ge X., Frank C.L., Madison J.M., Koehler A.N., Doud M.K., Tassa C., Berry E.M., Soda T., Singh K.K. (2009). Disrupted in schizophrenia 1 regulates neuronal progenitor proliferation via modulation of GSK3β/β-catenin signaling. Cell.

[92] Randall A.D., Kurihara M., Brandon N.J., Brown J.T. (2014). Disrupted in schizophrenia 1 and synaptic function in the mammalian central nervous system. Eur. J. Neurosci..

[93] Kim J.Y., Duan X., Liu C.Y., Jang M.H., Guo J.U., Pow-anpongkul N., Kang E., Song H., Ming G.L. (2009). DISC1 regulates new neuron development in the adult brain via modulation of AKT-mTOR signaling through KIAA1212. Neuron.

[94] Murdoch H., Mackie S., Collins D.M., Hill E.V., Bolger G.B., Klussmann E., Porteous D.J., Millar J.K., Houslay M.D. (2007). Isoformselective susceptibility of DISC1/phosphodiesterase-4 complexes to dissociation by elevated intracellular cAMP levels. J. Neurosci..

[95] Kim J.Y., Liu C.Y., Zhang F., Duan X., Wen Z., Song J., Feighery E., Lu B., Rujescu D., St Clair D. (2012). Interplay between DISC1 and GABA signaling regulates neurogenesis in mice and risk for schizophrenia. Cell.

[96] Wei J., Graziane N.M., Gu Z., Yan Z. (2015). DISC1 protein regulates γ-aminobutyric acid, type A (GABAA) receptor trafficking and inhibitory synaptic transmission in cortical neurons. J. Biol. Chem..

[97] Levchenko A., Davtian S., Freylichman O., Zagrivnaya M., Kostareva A., Malashichev Y. (2015). Betacatenin in schizophrenia: Possibly deleterious novel mutation. Psychiatry Res..

[98] Tang H., Shen N., Jin H., Liu D., Miao X., Zhu L.Q. (2013). GSK-3beta polymorphism discriminates bipolar disorder and schizophrenia: A systematic meta-analysis. Mol. Neurobiol..

[99] Tucci V., Kleefstra T., Hardy A., Heise I., Maggi S., Willemsen M.H., Hilton H., Esapa C., Simon M., Buenavista M.T. (2014). Dominant beta-catenin mutations cause intellectual disability with recognizable syndromic features. J. Clin. Invest..

[100] Balczon R., Bao L., Zimmer W.E. (1994). PCM-1, A 228-kD centrosome autoantigen with a distinct cell cycle distribution. J. Cell Biol..

[101] Kubo A., Sasaki H., Yuba-Kubo A., Tsukita S., Shiina N. (1999). Centriolar satellites: Molecular characterization, Atp-dependent movement toward centrioles and possible involvement in ciliogenesis. J. Cell Biol..

[102] Kubo A., Tsukita S. (2003). Non-membranous granular organelle consisting of PCM-1: Subcellular distribution and cell-cycle-dependent assembly/disassembly. J. Cell Sci..

[103] Vladar E.K., Stearns T. (2007). Molecular characterization of centriole assembly in ciliated epithelial cells. J. Cell Biol..

[104] Stowe T.R., Wilkinson C.J., Iqbal A., Stearns T. (2012). The centriolar satellite proteins Cep72 and Cep290 interact and are required for recruitment of BBS proteins to the cilium. Mol. Biol. Cell.

[105] Kim J., Krishnaswami S.R., Gleeson J.G. (2008). CEP290 interacts with the centriolar satellite component PCM-1 and is required for Rab8 localization to the primary cilium. Hum. Mol. Genet..

[106] Keryer G., Pineda J.R., Liot G., Kim J., Dietrich P., Benstaali C., Smith K., Cordelières F.P., Spassky N., Ferrante R.J. (2011). Ciliogenesis is regulated by a huntingtin-HAP1-PCM1 pathway and is altered in Huntington disease. J. Clin. Invest..

[107] Lopes C.A., Prosser S.L., Romio L., Hirst R.A., O′Callaghan C., Woolf A.S., Fry A.M. (2011). Centriolar satellites are assembly points for proteins implicated in human ciliopathies, including oral-facial-digital syndrome 1. J. Cell Sci..

[108] Eastwood S.L., Hodgkinson C.A., Harrison P.J. (2009). DISC-1 Leu607Phe alleles differentially affect centrosomal PCM1 localization and neurotransmitter release. Mol. Psychiatry.

[109] Eastwood S.L., Walker M., Hyde T.M., Kleinman J.E., Harrison P.J. (2010). The DISC1 Ser704Cys substitution affects centrosomal localisation of its binding partner PCM1 in glia in human brain. Hum. Mol. Genet..

[110] Gurling H.M., Critchley H., Datta S.R., McQuillin A., Blaveri E., Thirumalai S., Pimm J., Krasucki R., Kalsi G., Quested D. (2006). Genetic association and brain morphology studies and the chromosome 8p22 pericentriolar material 1 (PCM1) gene in susceptibility to schizophrenia. Arch. Gen. Psychiatry..

[111] Datta S.R., McQuillin A., Rizig M., Blaveri E., Thirumalai S., Kalsi G., Lawrence J., Bass N.J., Puri V., Choudhury K. (2010). A threonine to isoleucine missense mutation in the pericentriolar material 1 gene is strongly associated with schizophrenia. Mol. Psychiatry.

[112] Hashimoto R., Ohi K., Yasuda Y., Fukumoto M., Yamamori H., Kamino K., Morihara T., Iwase M., Kazui H., Numata S. (2011). No association between the PCM1 gene and schizophrenia: A multi-center case-control study and a meta-analysis. Schizophr. Res..

[113] Sakamoto S., Takaki M., Okahisa Y., Mizuki Y., Kodama M., Ujike H., Uchitomi Y. (2014). Four polymorphisms of the pericentriolar material 1 (PCM1) gene are not associated with schizophrenia in a Japanese population. Psychiatry Res..

[114] Zoubovsky S., Oh E.C., Cash-Padgett T., Johnson A.W., Hou Z., Mori S., Gallagher M., Katsanis N., Sawa A., Jaaro-Peled H. (2015). Neuroanatomical and behavioral deficits in mice haploinsufficient for Pericentriolar material 1 (Pcm1). Neurosci. Res..

[115] Jurczyk A., Gromley A., Redick S., Agustin J.S., Witman G., Pazour G.J., Peters D.J., Doxsey S. (2004). Pericentrin forms a complex with intraflagellar transport proteins and polycystin-2 and is required for primary cilia assembly. J. Cell Biol..

[116] Anitha A., Nakamura K., Yamada K., Iwayama Y., Toyota T., Takei N., Iwata Y., Suzuki K., Sekine Y., Matsuzaki H. (2008). Gene and expression analyses reveal enhanced expression of pericentrin 2 (PCNT2) in bipolar disorder. Biol. Psychiatry.

[117] Anitha A., Nakamura K., Yamada K., Iwayama Y., Toyota T., Takei N. (2009). Association studies and gene expression analyses of the DISC1-interacting molecules, pericentrin 2 (PCNT2) and DISC1—Binding zinc finger protein (DBZ), with schizophrenia and with bipolar disorder. Am. J. Med. Genet..

[118] Numata S., Nakataki M., Iga J.I., Tanahashi T., Nakadoi Y., Ohi K., Iwata Y., Suzuki K., Sekine Y., Matsuzaki H. (2010). Association study between the pericentrin (PCNT) gene and schizophrenia. Neuromol. Med..

[119] Miyoshi K., Asanuma M., Miyazaki I., Diaz-Corrales F.J., Katayama T., Tohyama M., Ogawa N. (2004). DISC1 localizes to the centrosome by binding to kendrin. Biochem. Biophys. Res. Commun..

[120] Poelmans G., Engelen J.J.M., Lent-Albrechts V., Smeets H.J., Schoenmakers E., Franke B., Buitelaar J.K., Wuisman-Frerker M., Erens W., Steyaert J. (2009). Identification of novel dyslexia candidate genes through the analysis of a chromosomal deletion. Am. J. Med. Genet..

[121] Ferland R.J., Eyaid W., Collura R.V., Tully L.D., Hill R.S., Al-Nouri D., Al-Rumayyan A., Topcu M., Gascon G., Bodell A. (2004). Abnormal cerebellar development and axonal decussation due to mutations in AHI1 in Joubert syndrome. Nature Genet..

[122] Dixon-Salazar T., Silhavy J.L., Marsh S.E., Louie C.M., Scott L.C., Gururaj A., Al-Gazali L., Al-Tawari A.A., Kayserili H., Sztriha L. (2004). Mutations in the AHI1 gene, encoding jouberin, cause Joubert syndrome with cortical polymicrogyria. Am. J. Hum. Genet..

[123] Louie C.M., Caridi G., Lopes V.S., Brancati F., Kispert A., Lancaster M.A., Schlossman A.M., Otto E.A., Leitges M., Gröne H.J. (2010). AHI1 is required for outer segment development and is a modifier for retinal degeneration in nephronophthisis. Nature Genet..

[124] Lee J.H., Gleeson J.G. (2010). The role of primary cilia in neuronal function. Neurobiol. Dis..

[125] Lancaster M.A., Schroth J., Gleeson J.G. (2011). Subcellular spatial regulation of canonical Wnt signaling at the primary cilium. Nature Cell Biol..

[126] Sheng G., Xu X., Lin Y.-F., Wang C.-E., Rong J., Cheng D., Peng J., Jiang X., Li S.H., Li X.J. (2008). Huntingtin-associated protein 1 interacts with Ahi1 to regulate cerebellar and brainstem development in mice. J. Clin. Invest..

[127] Eley L., Gabrielides C., Adams M., Johnson C.A., Hildebrandt F., Sayer J.A. (2008). Jouberin localizes to collecting ducts and interacts with nephrocystin-1. Kidney Int..

[128] Han S.B., Choi B.I., Lee D., Kee S.H., Kim H.S., Sun W., Kim H. (2009). Regulation of AHI1 expression in adult rat brain: Implication in hypothalamic feeding control. Biochem. Biophys. Res. Commun..

[129] Juric-Sekhar G., Adkins J., Doherty D., Hevner R.F. (2012). Joubert syndrome: brain and spinal cord malformations in genotyped cases and implications for neurodevelopmental functions of primary cilia. Acta Neuropathol..

[130] Amann-Zalcenstein D., Avidan N., Kanyas K., Ebstein R.P., Kohn Y., Hamdan A., Ben-Asher E., Karni O., Mujaheed M., Segman R.H. (2006). AHI1, a pivotal neurodevelopmental gene, and C6orf217 are associated with susceptibility to schizophrenia. Eur. J. Hum. Genet..

[131] Ingason A., Giegling I., Cichon S., Hansen T., Rasmussen H.B., Nielsen J., Jürgens G., Muglia P., Hartmann A.M., Strengman E. (2010). A large replication study and meta-analysis in European samples provides further support for association of AHI1 markers with schizophrenia. Hum. Mol. Genet..

[132] Ingason A., Sigmundsson T., Steinberg S., Sigurdsson E., Haraldsson M., Magnusdottir B.B., Frigge M.L., Kong A., Gulcher J., Thorsteinsdottir U. (2007). Support for involvement of the AHI1 locus in schizophrenia. Eur. J. Hum. Genet..

[133] Rivero O., Reif A., Sanjuan J., Molto M.D., Kittel-Schneider S., Najera C., Toepner T., Lesch K.P. (2010). Impact of the AHI1 gene on the vulnerability to schizophrenia: A case-control association study. PLoS ONE.

[134] Porcelli S., Pae C.-U., Han C., Lee S.-J., Patkar A.A., Masand P.S., Balzarro B., Alberti S., De Ronchi D., Serretti A. (2015). The influence of AHI1 variants on the diagnosis and treatment outcome in schizophrenia. Int. J. Mol. Sci..

[135] Slonimsky A., Levy I., Kohn Y., Rigbi A., Ben-Asher E., Lancet D., Agam G., Lerer B. (2010). Lymphoblast and brain expression of AHI1 and the novel primate-specific gene, C6orf217, in schizophrenia and bipolar disorder. Schizophr. Res..

[136] van Slegtenhorst M., de Hoogt R., Hermans C., Nellist M., Janssen B., Verhoef S., Lindhout D., Van den Ouweland A., Halley D., Young J. (1997). Identification of the tuberous sclerosis gene TSC1 on chromosome 9q34. Science.

[137] Benvenuto G., Li S., Brown S.J., Braverman R., Vass W.C., Cheadle J.P., Halley D.J., Sampson J.R., Wienecke R., DeClue J.E. (2000). The tuberous sclerosis-1 (TSC1) gene product hamartin suppresses cell growth and augments the expression of the TSC2 product tuberin by inhibiting its ubiquitination. Oncogene.

[138] Inoki K., Li Y., Zhu T., Wu J., Guan K.L. (2002). TSC2 is phosphorylated and inhibited by Akt and suppresses mTOR signalling. Nat. Cell Biol..

[139] Kwiatkowski D.J., Manning B.D. (2005). Tuberous sclerosis: A GAP at the crossroads of multiple signaling pathways. Hum. Mol. Genet..

[140] Astrinidis A., Senapedis W., Henske E.P. (2006). Hamartin, the tuberous sclerosis complex 1 gene product, interacts with polo-like kinase 1 in a phosphorylation-dependent manner. Hum. Mol. Genet..

[141] Bonnet C.S., Aldred M., von Ruhland C., Harris R., Sandford R., Cheadle J.P. (2009). Defects in cell polarity underlie TSC and ADPKD-associated cystogenesis. Hum. Mol. Genet..

[142] Kwon C.-H., Luikart B.W., Powell C.M., Zhou J., Matheny S.A., Zhang W., Li Y., Baker S.J., Parada L.F. (2006). Pten regulates neuronal arborization and social interaction in mice. Neuron.

[143] Ehninger D., de Vries P.J., Silva A.J. (2009). From mTOR to cognition: Molecular and cellular mechanisms of cognitive impairments in tuberous sclerosis. J. Intellect. Disabil. Res..

[144] Gomez M.R. (1991). Phenotypes of the tuberous sclerosis complex with a revision of diagnostic criteria. Ann. N. Y. Acad. Sci..

[145] Wataya-Kaneda M. (2015). Mammalian target of rapamycin and tuberous sclerosis complex. J. Dermatol. Sci..

[146] Tsai P.T., Chu Y., Greene-Colozzi E., Sadowski A.R., Leech J.M., Steinberg J., Crawley J.N., Regehr W.G., Sahin M. (2012). Autistic-like behaviour and cerebellar dysfunction in Purkinje cell Tsc1 mutant mice. Nature.

[147] Mak B.C., Takemaru K.I., Kenerson H.L., Moon R.T., Yeung R.S. (2003). The tuberin-hamartin complex negatively regulates β-catenin signaling activity. J. Biol. Chem..

[148] Kalkman H.O. (2012). A review of the evidence for the canonical Wnt pathway in autism spectrum disorders. Mol. Autism.

[149] Meng H., Smith S.D., Hager K., Held M., Liu J., Olson R.K., Pennington B.F., DeFries J.C., Gelernter J., O′Reilly-Pol T. (2005). DCDC2 is associated with reading disability and modulates neuronal development in the brain. Proc. Natl. Acad. Sci. USA.

[150] Schumacher J., Anthoni H., Dahdouh F., Konig I.R., Hillmer A.M., Kluck N., Manthey M., Plume E., Warnke A., Remschmidt H. (2006). Strong genetic evidence of DCDC2 as a susceptibility gene for dyslexia. Am. J. Hum. Genet..

[151] Schueler M., Braun D.A., Chandrasekar G., Gee H.Y., Klasson T.D., Halbritter J., Bieder A., Porath J.D., Airik R., Zhou W. (2015). DCDC2 Mutations Cause a Renal-Hepatic Ciliopathy by Disrupting Wnt Signaling. Am. J. Hum. Genet..

[152] Grati M.H., Chakchouk I., Ma Q., Bensaid M., Desmidt A., Turki N., Yan D., Baanannou A., Mittal R., Driss N. (2015). A missense mutation in DCDC2 causes human recessive deafness DFNB66, likely by interfering with sensory hair cell and supporting cell cilia length regulation. Hum. Mol. Genet..

[153] Marino C., Meng H., Mascheretti S., Rusconi M., Cope N., Giorda R., Molteni M., Gruen J.R. (2012). DCDC2 genetic variants and susceptibility to developmental dyslexia. Psychiatric Genetics.

[154] Harold D., Paracchini S., Scerri T., Dennis M., Cope N., Hill G., Moskvina V., Walter J., Richardson A.J., Owen M.J. (2006). Further evidence that the KIAA0319 gene confers susceptibility to developmental dyslexia. Mol. Psychiatry.

[155] Jamadar S., Powers N.R., Meda S.A., Gelernter J., Gruen J.R., Pearlson G.D. (2011). Genetic influences of cortical grey matter in language-related regions in healthy controls and schizophrenia. Schizophrenia Res..

[156] Darki F., Peyrard-Janvid M., Matsson H., Kere J., Klingberg T. (2012). Three dyslexia susceptibility genes, DYX1C1, DCDC2, and KIAA0319, affect temporo-parietal white matter structure. Biol. Psychiatry.

[157] Taipale M., Kaminen N., Nopola-Hemmi J., Haltia T., Myllyluoma B., Lyytinen H., Muller K., Kaaranen M., Lindsberg P.J., Hannula-Jouppi K. (2003). A candidate gene for developmental dyslexia encodes a nuclear tetratricopeptide repeat domain protein dynamically regulated in brain. Proc. Natl. Acad. Sci. USA.

[158] Ivliev A.E., AC′t Hoen P.A.C., van Roon-Mom W.M., Peters D.J., Sergeeva M.G. (2012). Exploring the transcriptome of ciliated cells using in silico dissection of human tissues. PLoS ONE.

[159] Hoh R.A., Stowe T.R., Turk E., Stearns T. (2012). Transcriptional rogram of ciliated epithelial cells reveals new cilium and centrosome components and links to human disease. PLoS ONE.

[160] Lim C.K., Ho C.S., Chou C.H., Waye M.M. (2011). Association of the rs3743205 variant of DYX1C1 with dyslexia in Chinese children. Behav. Brain Funct..

[161] Paracchini S., Ang Q.W., Stanley F.J., Monaco A.P., Pennell C.E., Whitehouse A.J.O. (2011). Analysis of dyslexia candidate genes in the Raine cohort representing the general Australian population. Genes Brain Behav..

[162] Venkatesh S.K., Siddaiah A., Padakannaya P., Ramachandra N.B. (2014). Association of SNPs of DYX1C1 with developmental dyslexia in an Indian population. Psychiatric Genet..

[163] Rendall A.R., Tarkar A., Contreras-Mora H.M., LoTurco J.J., Fitch R.H. (2015). Deficits in learning and memory in mice with a mutation of the candidate dyslexia susceptibility gene Dyx1c1. Brain Lang..

[164] Badano J.L., Mitsuma N., Beales P.L., Katsanis N. (2006). The ciliopathies: An emerging class of human genetic disorders. Annu. Rev. Genomics Hum. Genet..

[165] Norris D.P., Grimes D.T. (2012). Mouse models of ciliopathies: The state of the art. Dis. Model. Mech..

[166] Harrison P.J. (1999). The neuropathology of schizophrenia. Brain.

[167] Wegiel J., Kuchna I., Nowicki K., Imaki H., Wegiel J., Marchi E., Ma S.Y., Chauhan A., Chauhan V., Bobrowicz T.W. (2010). The neuropathology of autism: Defects of neurogenesis and neuronal migration, and dysplastic changes. Acta Neuropathol..

[168] Tabares-Seisdedos R., Escamez T., Martinez-Gimenez J.A., Balanza V., Salazar J., Selva G., Rubio C., Vieta E., Geijo-Barrientos E., Martinez-Aran A. (2006). Variations in genes regulating neuronal migration predict reduced prefrontal cognition in schizophrenia and bipolar subjects from mediterranean Spain: A preliminary study. Neuroscience.

[169] Galaburda A.M., Sherman G.F., Rosen G.D., Abolitz F., Geschwind N. (1985). Developmental dyslexia: Four consecutive patients with cortical anomalies. J. Neurol..

[170] Beasley C.L., Cotter D.R., Everall I.P. (2002). Density and distribution of white matter neurons in schizophrenia, bipolar disorder and major depressive disorder: No evidence for abnormalities of neuronal migration. Mol. Psychiatry.

[171] Kuijpers M., Hoogenraad C.C. (2011). Centrosomes, microtubules and neuronal development. Mol. Cell. Neurosci..

[172] Delaval B., Doxsey S.J. (2010). Pericentrin in cellular function and disease. J. Cell Biol..

[173] Guadiana S.M., Semple-Rowland S., Daroszewski D., Madorsky I., Breunig J.J., Mykytyn K., Sarkisian M.R. (2013). Arborization of dendrites by developing neocortical neurons is dependent on primary cilia and type 3 adenylyl cyclase. J. Neurosci..

[174] Hayashi-Takagi A., Takaki M., Graziane N., Seshadri S., Murdoch H., Dunlop A.J., Makino Y., Seshadri A.J., Ishizuka K., Srivastava D.P. (2010). Disrupted-in-Schizophrenia-1 (DISC1) regulates spines of the glutamate synapse via Rac1. Nature Neurosci..

[175] Ross C.A., Margolis R.L., Reading S.A., Pletnikov M., Coyle J.T. (2006). Neurobiology of schizophrenia. Neuron.

[176] Schmitt A., Hasan A., Gruber O., Falkai P. (2011). Schizophrenia as a disorder of disconnectivity. Eur. Archives Psychiatry Clin. Neurosci..

[177] Brambilla P., Hardan A., di Nemi S.U., Perez J., Soares J.C., Barale F. (2003). Brain anatomy and development in autism: Review of structural MRI studies. Brain Res. Bull..

[178] Fu C., Cawthon B., Clinkscales W., Bruce A., Winzenburger P., Ess K.C. (2012). GABAergic interneuron development and function is modulated by the Tsc1 gene. Cereb. Cortex.

[179] Twelvetrees A.E., Yuen E.Y., Arancibia-Carcamo I.L., MacAskill A.F., Rostaing P., Lumb M.J., Humbert S., Triller A., Saudou F., Yan Z. (2010). Delivery of GABAARS to synapses is mediated by HAP1-KIF5 and disrupted by mutant huntingtin. Neuron.

[180] Currier T.A., Etchegaray M.A., Haight J.L., Galaburda A.M., Rosen G.D. (2011). The effects of embryonic knockdown of the candidate dyslexia susceptibility gene homologue Dyx1c1 on the distribution of GABAergic neurons in the cerebral cortex. Neurosci. Biobehav. Rev..

[181] Ge S., Pradhan D.A., Ming G.L., Song H. (2007). GABA sets the tempo for activity-dependent adult neurogenesis. Trends Neurosci..

[182] Petryshen T.L., Middleton F.A., Tahl A.R., Rockwell G.N., Purcell S., Aldinger K.A., Kirby A., Morley C.P., McGann L., Gentile K.L. (2005). Genetic investigation of chromosome 5q GABAA receptor subunit genes in schizophrenia. Mol. Psychiatry.

[183] Lewis D.A., Volk D.W., Hashimoto T. (2004). Selective alterations in prefrontal cortical GABA neurotransmission in schizophrenia: A novel target for the treatment of working memory dysfunction. Psychopharmacology.

[184] Benes F.M., Berretta S. (2001). GABAergic interneurons: Implications for understanding schizophrenia and bipolar disorder. Neuropsychopharmacology.

[185] Coghlan S., Horder J., Inkster B., Mendez M.A., Murphy D.G., Nutt D.J. (2012). GABA system dysfunction in autism and related disorders: From synapse to symptoms. Neurosci. Biobehav. Rev..

[186] Breunig J.J., Sarkisian M.R., Arellano J.I., Morozov Y.M., Ayoub A.E., Sojitra S., Wang B., Flavell R.A., Rakic P., Town T. (2008). Primary cilia regulate hippocampal neurogenesis by mediating sonic hedgehog signaling. Proc. Natl. Acad. Sci. USA.

[187] Lie D.C., Colamarino S.A., Song H.J., Desire L., Mira H., Consiglio A., Lein E.S., Jessberger S., Lansford H., Dearie A.R. (2005). Wnt signaling regulates adult hippocampal neurogenesis. Nature.

[188] Freyberg Z., Ferrando S.J., Javitch J.A. (2009). Roles of the Akt/GSK-3 and Wnt signaling pathways in schizophrenia and antipsychotic drug action. Am. J. Psychiatry.

[189] Fu H., Subramanian R.R., Masters S.C. (2000). 14–3-3 proteins: Structure, function, and regulation. Annu. Rev. Pharmacol. Toxicol..

[190] Bruno D.L., Anderlid B.M., Lindstrand A., van Ravenswaaij-Arts C., Ganesamoorthy D., Lundin J., Martin C.L., Douglas J., Nowak C., Adam M.P. (2010). Further molecular and clinical delineation of co-locating 17p13. 3 microdeletions and microduplications that show distinctive phenotypes. J. Med. Genet..

[191] Capra V., Mirabelli-Badenier M., Stagnaro M., Rossi A., Tassano E., Gimelli S., Gimelli G. (2012). Identification of a rare 17p13.3 duplication including the BHLHA9 and YWHAE genes in a family with developmental delay and behavioural problems. BMC Med. Genet..

[192] Curry C.J., Rosenfeld J.A., Grant E., Gripp K.W., Anderson C., Aylsworth A.S., Saad T.B., Chizhikov V.V., Dybose G., Fagerberg C. (2013). The duplication 17p13. 3 phenotype: Analysis of 21 families delineates developmental, behavioral and brain abnormalities, and rare variant phenotypes. Am. J. Med. Genet..

[193] Grover D., Verma R., Goes F.S., Mahon P.L.B., Gershon E.S., McMahon F.J., Potash J.B. (2009). Family based association of YWHAH in psychotic bipolar disorder. Am. J. Med. Genet..

[194] Liu J., Li Z.Q., Li J.Y., Li T., Wang T., Li Y., Xu Y.F., Feng G.Y., Shi Y.Y., He L. (2012). Polymorphisms and haplotypes in the YWHAE gene increase susceptibility to bipolar disorder in Chinese Han population. J. Clin. Psychiatry.

[195] Kido M., Nakamura Y., Nemoto K., Takahashi T., Aleksic B., Furuichi A., Nakamura Y., Ikeda M., Noguchi K., Kaibuchi K. (2014). The Polymorphism of YWHAE, a Gene Encoding 14–3-3Epsilon, and Brain Morphology in Schizophrenia: A Voxel-Based Morphometric Study. PLoS ONE.

[196] Takahashi T., Nakamura Y., Nakamura Y., Aleksic B., Takayanagi Y., Furuichi A., Kido M., Nakamura M., Sasabayashi D., Ikeda M. (2014). The polymorphism of YWHAE, a gene encoding 14–3-3epsilon, and orbitofrontal sulcogyral pattern in patients with schizophrenia and healthy subjects. Prog. Neuropsychopharmacol. Biol. Psychiatry.

[197] Bunney T.D., De Boer A.H., Levin M. (2003). Fusicoccin signaling reveals 14–3-3 protein function as a novel step in left-right patterning during amphibian embryogenesis. Development.

[198] Nellist M., Goedbloed M.A., Halley D.J.J. (2003). Regulation of tuberous sclerosis complex (TSC) function by 14–3-3 proteins. Biochem. Soc. Trans..

[199] Hengstschläger M., Rosner M., Fountoulakis M., Lubec G. (2003). Tuberous sclerosis genes regulate cellular 14–3-3 protein levels. Biochem.

[200] Cheah P.S., Ramshaw H.S., Thomas P.Q., Toyo-Oka K., Xu X., Martin S., Coyle P., Guthridge M.A., Stomski F., Van Den Buuse M. (2012). Neurodevelopmental and neuropsychiatric behaviour defects arise from 14–3-3ζ deficiency. Mol. Psychiatry.

[201] Taya S., Shinoda T., Tsuboi D., Asaki J., Nagai K., Hikita T., Kuroda S., Kuroda K., Shimizu M., Hirotsune S. (2007). DISC1 regulates the transport of the NUDEL/LIS1/14–3-3ε complex through kinesin-1. J. Neurosci.

[202] Sherwood V., Manbodh R., Sheppard C., Chalmers A.D. (2008). RASSF7 is a member of a new family of RAS association domain–containing proteins and is required for completing mitosis. Mol. Biol. Cell.

[203] Vingerhoets G., Li X., Bogaert S., Roberts N. Hand preference, cognitive performance, and brain asymmetry in situs inversus totalis. Proceedings of North Sea Laterality Meeting 2016.

